# Development and Characterization of High Efficacy Cell-Penetrating Peptide via Modulation of the Histidine and Arginine Ratio for Gene Therapy

**DOI:** 10.3390/ma14164674

**Published:** 2021-08-19

**Authors:** Yu Liu, Huan-Huan Wan, Duo-Mei Tian, Xiao-Jun Xu, Chang-Long Bi, Xiao-Yong Zhan, Bi-Hui Huang, Yun-Sheng Xu, Le-Ping Yan

**Affiliations:** 1 Department of Dermatovenereology, The Seventh Affiliated Hospital, Sun Yat-sen University, Shenzhen 518107, China; liuy826@mail2.sysu.edu.cn (Y.L.); wanhh3@mail2.sysu.edu.cn (H.-H.W.); 2 Scientific Research Center, The Seventh Affiliated Hospital, Sun Yat-sen University, Shenzhen 518107, China; tiandm@mail2.sysu.edu.cn (D.-M.T.); zhanxy7@mail.sysu.edu.cn (X.-Y.Z.); huangbh7@mail.sysu.edu.cn (B.-H.H.); 3 Department of Emergency and Intensive Care Medicine, The Seventh Affiliated Hospital, Sun Yat-sen University, Shenzhen 518107, China; 4 Department of Hematology, The Seventh Affiliated Hospital, Sun Yat-sen University, Shenzhen 518107, China; xuxj29@mail.sysu.edu.cn; 5 Department of Endocrinology, The Eighth Affiliated Hospital, Sun Yat-sen University, Shenzhen 518033, China; BCL163@163.com; 6 Guangdong Provincial Key Laboratory of Digestive Cancer Research, The Seventh Affiliated Hospital, Sun Yat-sen University, Shenzhen 518107, China

**Keywords:** gene therapy, cell penetrating peptide, histidine, RALA peptide, endosomal escaping

## Abstract

Cell-penetrating peptides (CPPs), as non-viral gene delivery vectors, are considered with lower immunogenic response, and safer and higher gene capacity than viral systems. In our previous study, a CPP peptide called RALA (arginine rich) presented desirable transfection efficacy and owns a potential clinic use. It is believed that histidine could enhance the endosome escaping ability of CPPs, yet RALA peptide contains only one histidine in each chain. In order to develop novel superior CPPs, by using RALA as a model, we designed a series of peptides named HALA (increased histidine ratio). Both plasmid DNA (pDNA) and siRNA transfection results on three cell lines revealed that the transfection efficacy is better when histidine replacements were on the C-terminal instead of on the N-terminal, and two histidine replacements are superior to three. By investigating the mechanism of endocytosis of the pDNA nanocomplexes, we discovered that there were multiple pathways that led to the process and caveolae played the main role. During the screening, we discovered a novel peptide-HALA2 of high cellular transfection efficacy, which may act as an exciting gene delivery vector for gene therapy. Our findings also bring new insights on the development of novel robust CPPs.

## 1. Introduction

Since Friedmann and Roblin firstly proposed the concept of gene therapy in 1972, it became a promising therapeutic option with a great potential to treat many genetic and acquired diseases [1,2,3]. The primal principle of gene therapy is to introduce foreign genetic material into host cells via suitable vectors, in order to promote the expression of therapeutic proteins or to silence the relevant genes. Several strategies have already been tested in clinical trials, and the used nucleic acids include DNA, message RNA (mRNA), micro RNA (miRNA), short interfering RNA (siRNA), short hairpin RNA (shRNA), small activating RNA (saRNA) and antisense oligonucleotides (ASO), and even patient-derived cellular gene therapy [4,5,6,7,8,9,10,11].

As for a delivery platform, viral and non-viral gene delivery systems have been developed and studied through the past several decades, and the utility of these two systems has been greatly investigated. Non-viral gene delivery systems, e.g., liposomes, cationic polymers, and cell-penetrating peptides (CPPs), are considered with properties of lower immunogenic response, safety, higher gene capacity, more stability and more flexibility of chemical design than viral systems [12]. CPPs typically have 5–30 amino acids and possess the ability to cross the cell membrane. Since Frankel and Pabo discovered the first CPP, trans-activator of transcription (TAT) peptide in 1988, which was originally encoded by human immunodeficiency virus type 1 (HIV-1), there are already more than 1700 CPPs that have been discovered or made nowadays [13,14]. CPPs have a variety of applications, such as acting as vectors for nucleic acid condensation, incorporation of functional motif [15,16,17], or even for anti-microbial application [18,19].

The RALA peptide (N-WEARLARALARALARHLARALARALRACEA-C) was originated from fusogenic peptides GALA (glutamic acid rich) and KALA (lysine rich), and first reported by McCarthy et al. in 2014 [20]. It was artificially designed from the KALA peptide by replacing all the lysine residues in KALA by arginine residues, presenting infinite possibilities for nucleic acid therapeutics [20]. It has been used in dissolving microneedles for DNA vaccination [21], or condensation of DNA into nanoparticles to be encapsulated within polylactic acid–polyethylene glycol copolymers [22]. Our team recently collaborated with McCarthy et al. and developed collagen/glycosaminoglycan (GAG) scaffolds activated by RALA complexed matrix metalloproteinase-9 siRNA (siRNA-MMP-9) for improving diabetic foot ulcer healing by reducing the MMP-9 expression from fibroblast [23].

Histidine is an amino acid that has an imidazole group on the side chain with the pKa value of 6.0, which is lower than that of lysine (pKa 10.53) or arginine (pKa 12.48). Histidine-rich peptides are usually considered as being endosomolytic in nature, as it can accelerate the endosomal escaping process through the proton sponge phenomenon or “flip-flop” effects [24,25]. One example of histidine application in CPP was the modification of wild-type S4(13)-PV by adding a five-histidine tail to its N-terminus, resulting in the significantly improved efficacy of peptide-mediated gene silencing in human fibrosarcoma HT1080 cells [26]. The high transfection efficacy of RALA was mainly due to the arginine in its backbone. Considering RALA has only one histidine in its backbone, in order to unlock the transfection potential of RALA, we aim to develop more advanced CPPs by modulation the histidine and arginine ratio in RALA. Inspired by the evolution history of GALA, KALA and RALA peptides, we designed a series of peptides named HALA (Table 1). Dependent on the numbers and positions of histidine replacements on the sequence of RALA peptide, we named them HALA1 to HALA4 respectively. HALA1 and HALA2 replace two arginine by histidine on the N-terminal and C-terminal of the sequence respectively, while HALA3 and HALA4 have three replacements on the N-terminal and C-terminal respectively. In this study, we designed HALA1, HALA2, HALA3, and HALA4 for comprehensive in vitro gene transfection study, due to their representativeness of replacing the arginine number (two and three arginine were replaced) and positions of replaced arginine on the peptide sequence (N-terminal and C-terminal).

## 2. Results

### 2.1. Physicochemical Characterization and Cytocompatibility Evaluation of Gene Nanocomplexes

**Figure 1:**
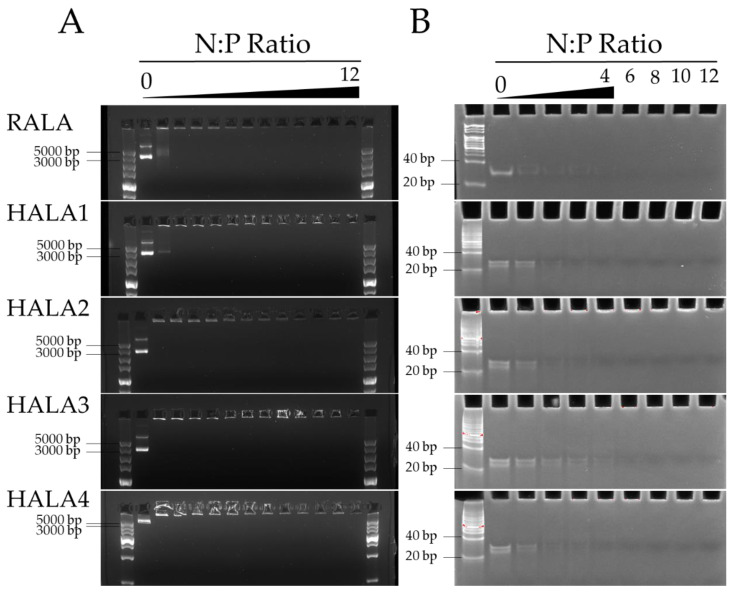
The gel retardation of nanocomplexes. (**A**) Gel retardation assay of pDNA and peptide nanocomplexes. Nanoparticles were complexed by pDNA and HALA series peptides and RALA peptide. N:P ratios 0–12 were tested. The nanocomplexes were loaded into a 1% agarose gel which was subsequently run within an electrophoresis field of 120 V for 30 min. (**B**) Gel retardation assay of siRNA and peptide nanocomplexes. Nanoparticles were complexed by non-target siRNA and HALA series peptides and RALA peptide. N:P ratios 0–4, 6, 8, 10, 12 were tested. The nanocomplexes were loaded into a 20% polyacrylamide gel which was run in an electrophoresis field of 80 V for 120 min.

We generated the nanoparticles in various N:P ratios, the molar ratio of positively charged nitrogen atoms in the peptide to negatively charged phosphate in the nucleic acid backbone, complexed by plasmid DNA (pDNA) (5010 bp) and siRNA (24 bp) with RALA and HALA1, HALA2, HALA3, and HALA4 peptides. The gel retardation results indicated that the HALA series peptides were fully capable in condensing pDNA from N:P 2 onwards, by showing no extra DNA could be detected on the gel (Figure 1A).

Interestingly, we even observed that in HALA2, HALA3 and HALA4, the N:P ratio of 1 showed no extra DNA being visible on the gel. Meanwhile, pDNA not shown on the gel were retained within the wells of the gel. As for siRNA condensation, except HALA3 that presented stable condensation from N:P 6 onward, the rest of the peptides presented from N:P 4 onward (Figure 1B). This indicated that two and even three arginine replacements led to little impact on the condensational ability of the RALA peptide. All nanocomplexes presented a great condensation ability from N:P 6 onwards.

**Figure 2:**
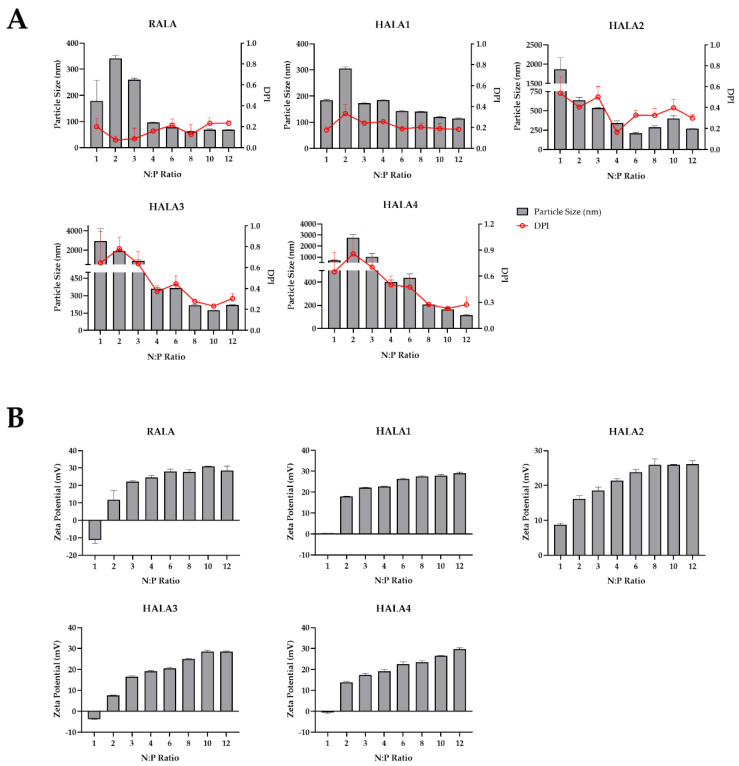
Particle size and zeta potential of nanocomplexes. Nanoparticles complexed by RALA or HALA series peptides and pDNA of N:P ratios of 1, 2, 3, 4, 6, 8, 10, 12 were created for detection of particle size with DPI (**A**) and zeta potential (**B**).

Next, we measured the mean hydrodynamic particle size and zeta potential of the nanocomplexes. The results indicated that all peptides were able to condense pDNA into a nanoparticle < 200 nm and contain a positive charge of around +25 mV from N:P 6 onward, within the range for optimal cellular uptake (Figure 2). This indicated again that comparing with the RALA peptide, two and even three arginine replacements result in little impact on the condensation of the RALA peptide and charge of the nanoparticle by the pDNA and peptides.

**Figure 3:**
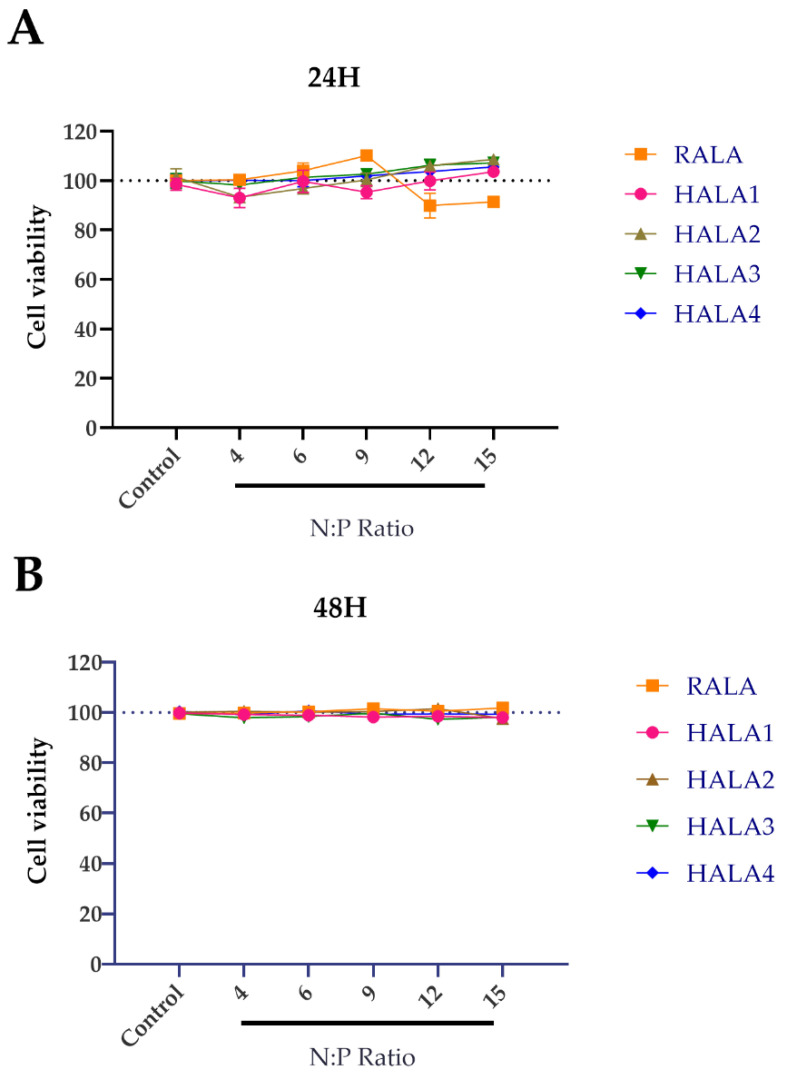
Cell viability post transfection. Percentages of cell viability of HeLa cells (**A**) 24 and (**B**) 48 h post transfection by RALA and HALA series peptides and pDNA complexes in N:P ratios of 4, 6, 9, 12, 15.

In order to detect whether the HALA series peptides are harmful to cells, we evaluated the cytocompatibility of nanoparticles by transfection of HeLa cell lines with varied nanocomplexes developed by complexing the peptides and pDNA in N:P ratios ranging from 4 to 15. The cell viability of HeLa cells was measured 24 and 48 h post-transfection. With no significant cell death compared to control detected (Figure 3), the results indicated that all nanoparticles were noncytotoxic and presented no harmful impact on cell proliferation.

Considering the promising result of nanocomplexes at N:P 6, we chose this N:P ratio for further in vitro experiments.

### 2.2. In Vitro Evaluation of the Cell Transfection Efficacy of the Nanocomplexes

**Figure 4:**
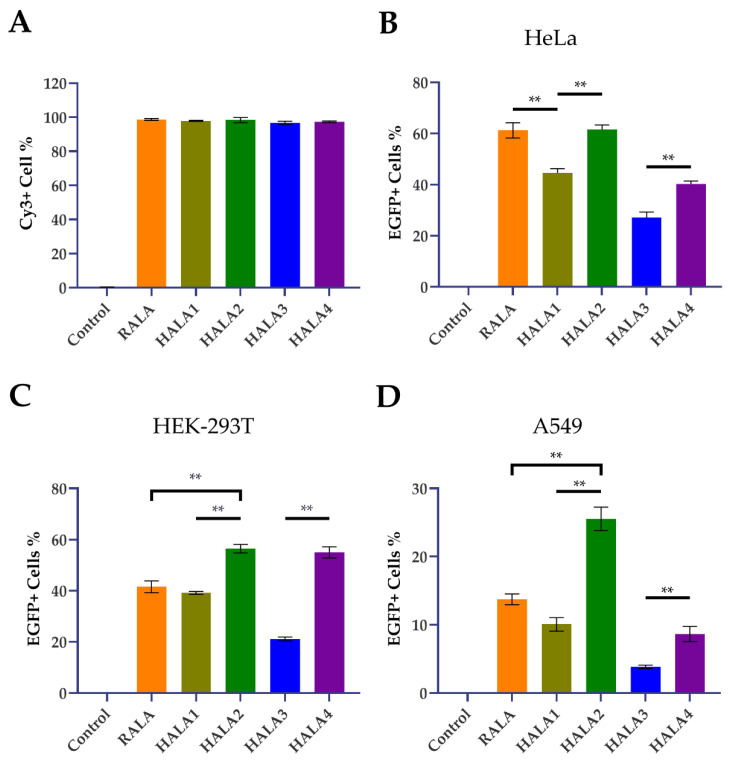
Cellular uptake and transfection efficacy of the nanoparticles. (**A**) Uptake efficiency of nanoparticles. (**B**–**D**) Transfection results of nanoparticles in HeLa cells (**B**), HEK-293T cells (**C**) and A549 cells (**D**). A group of cells treated with bare pDNA solution containing 1 μg per well of 24-well plate were used as control; ** indicates *p* < 0.01.

A 5′Cy3 modified non-target siRNA was used as reporter cargo to identify the nanocomplexes during the transfection process. As detected by flow cytometry, nanocomplexes generated by all these peptides showed high efficacy in cellular uptake (incubation for 6 h with nanocomplexes) with >97% Cy3 positive cells in all transfection groups (Figure 4A, Supplementary Figure S1A).

**Figure 5:**
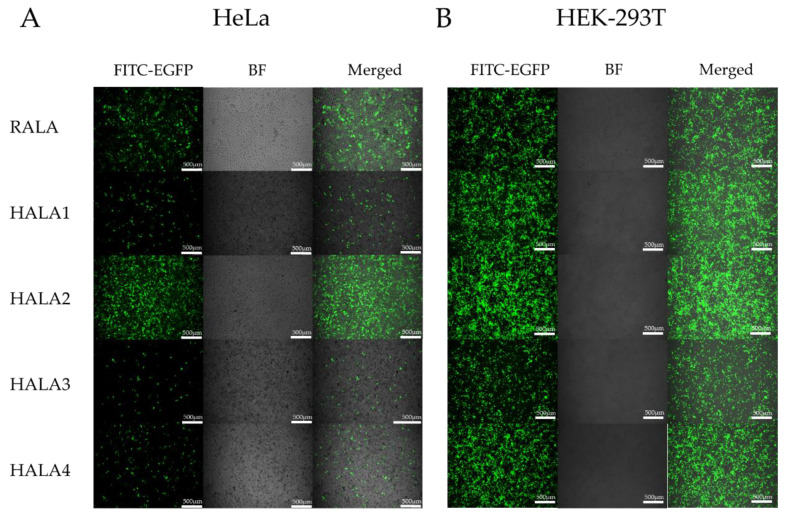
Fluorescent images of cells transfected with nanocomplexes. Fluorescent images (green, for detecting Enhanced Green Fluorescent Protein (EGFP) expression) and bright field images of HeLa cells (**A**) and HEK-293T cells (**B**) post transfection. Transfection nanocomplexes were generated by complexing the RALA and HALA series peptides with the pCMV-EGFP pDNA at a N:P ratio of 6.

Next, in order to evaluate the transfection ability of the HALA series and RALA peptides, we employed fluorescent microscopy and flow cytometric analysis to analyze the transfection efficacy of nanoparticles. Nanoparticles used in the transfection assay were complexed with RALA and HALA series peptides and pCMV-Enhanced Green Fluorescent Protein (EGFP) plasmid DNA with a N:P ratio of 6. Three different cell lines including normal and tumoral were selected. The first cell line we chose is a human cervical cancer cell line, HeLa, for the assessment of the transfection by nanoparticles. According to the results of fluorescent images in post transfected cells, HALA2 and RALA groups both gained a higher transfected efficacy compared toother groups (Figure 5A). Meanwhile, the flow cytometry data also demonstrated that the pDNA transfection efficacies of the RALA and HALA2 groups were quite similar, both above 60% (Figure 4B, Supplementary Figure S1B), with no statistical significance. Yet, the HALA1 group demonstrated less transfection efficacy which was around 45%, compared to RALA and HALA2 groups. Interestingly, the HALA3 and HALA4 groups showed inferior transfection efficacies to the RALA group, though they were designed with one more arginine replaced by histidine. Yet, while comparing the HALA1 and HALA2 groups, the results revealed that the transfection efficacies were better for the HALA2 group. The comparison results between HALA3 and HALA4 demonstrated a similar trend, i.e., the replacements of the same numbers of arginine with histidine on the C-terminal resulted in better transfection efficacy compared to the replacements on the N-terminal.

Next, another cell line, the human embryonic kidney (HEK)-293T cell, was used to further evaluate the transfection abilities of the peptides. Under the microscope, HALA3 seemed to present less Enhanced Green Fluorescent Protein (EGFP) positive cells compared to the other groups (Figure 5B). Told by flow cytometry, around 60% EGFP positive cells were detected in both HALA2 and HALA4 groups post-transfection. Yet HALA1 and RALA were showing similar values, which were around 40% (Figure 4C, Supplementary Figure S1C). HALA3 group presented the least value among the groups. We further measured the transfection abilities on another cell line A549, which is a lung non-small cell lung cancer cell line. HALA2 group, told by flow cytometry analysis, presented about 2 times of EGFP positive cells compared to RALA group (Figure 4D, Supplementary Figure S1D). HALA1 presented a bit lower transfection efficacy than the RALA group. Both HALA3 and HALA4 groups showed inferior transfection efficacy compared to HALA1. Similar to the observation in HeLa cells transfection, we also noticed the same trend in HEK-293T and A549 cells transfection that histidine replacements in RALA C-terminal induced higher transfection efficacy compared to the replacement in N-terminal.

**Figure 6:**
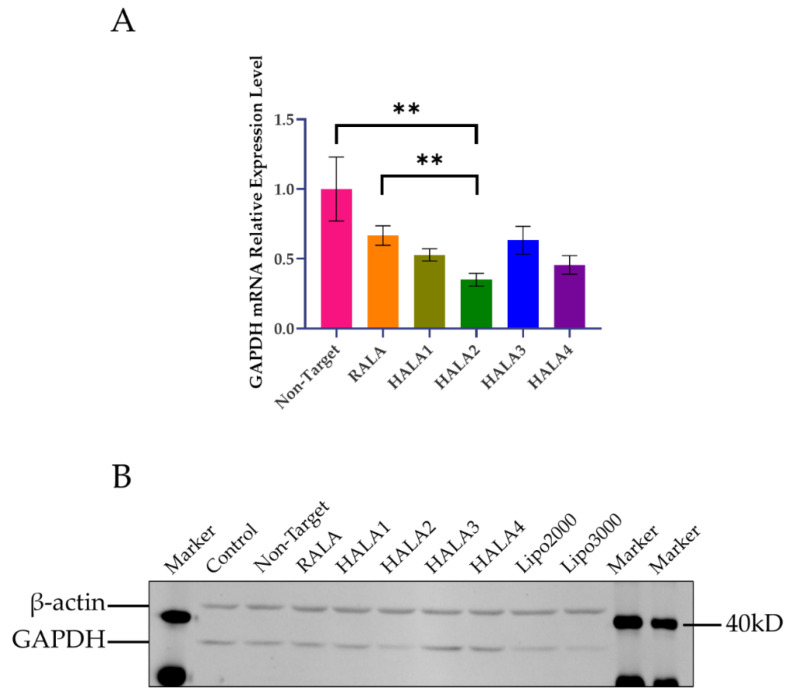
Gene silencing efficacy of nanocomplexes. (**A**) qRT-PCR analysis of the relative GAPDH mRNA expression level on A549 cells after transfection with GAPDH siRNA via the nanocomplexes. Actin was used as housekeeping gene. (**B**) GAPDH protein expression level presented by Western blotting. Cells without transfection and treated with non-target siRNA were used as controls. ** indicates *p* < 0.01.

After measuring pDNA delivery efficacy, we used siRNA-Glyceraldehyde-3-phosphate dehydrogenase (GAPDH) as our model siRNA to evaluate the difference between RALA and HALA series peptides in silencing gene. A549 cell line was employed for gene silencing in this experiment. Quantitative real-time polymerase chain reaction (qRT-PCR) analysis and Western blotting were performed to measure the silencing efficacy on the mRNA and protein levels of GAPDH gene. The qRT-PCR results showed that all the nanoparticles induced significant downregulation of GAPDH mRNA expression level (Figure 6A). HALA2 peptide presented the highest inhibition level among all groups. HALA1 and HALA3 reached almost the same level as RALA. Yet, we observed the same trend that happened in the pDNA transfection experiments, where the N-terminal replacement of arginine by histidine could cause an efficiency decrease in delivering nucleic acid. On the protein level, the Western blotting result made the downregulation of GAPDH more obvious (Figure 6B). The GAPDH protein expression levels on group HALA2, two groups of commercially available transfection reagent Lipofectamine^®^ 2000 and Lipofectamine^®^ 3000, were alike, all being lower than other groups. Other groups showed no significant difference. This result indicated that HALA2 is indeed a promising platform for siRNA delivery.

Collectively, our data suggest that the arginine replacement by histidine on RALA peptide on the C-terminal is capable of increasing the transfection efficacy without sacrificing the condensation or cellular uptake efficacy.

### 2.3. Transfection Mechanism of the Nanocomplexes

According to previous studies, the replacement of arginine by histidine is capable of increasing the efficacy of escaping from endosome [24,25]. Therefore, we employed chloroquine, a known endosomal disruptor, which is able to damage the formation process of endosome by inhibit its acidification. The transfection results were quantified by flow cytometry analysis as previous described. All nanoparticles were used at the N:P ratio of 6. The A549 cell line was employed for the upcoming investigation.

**Figure 7:**
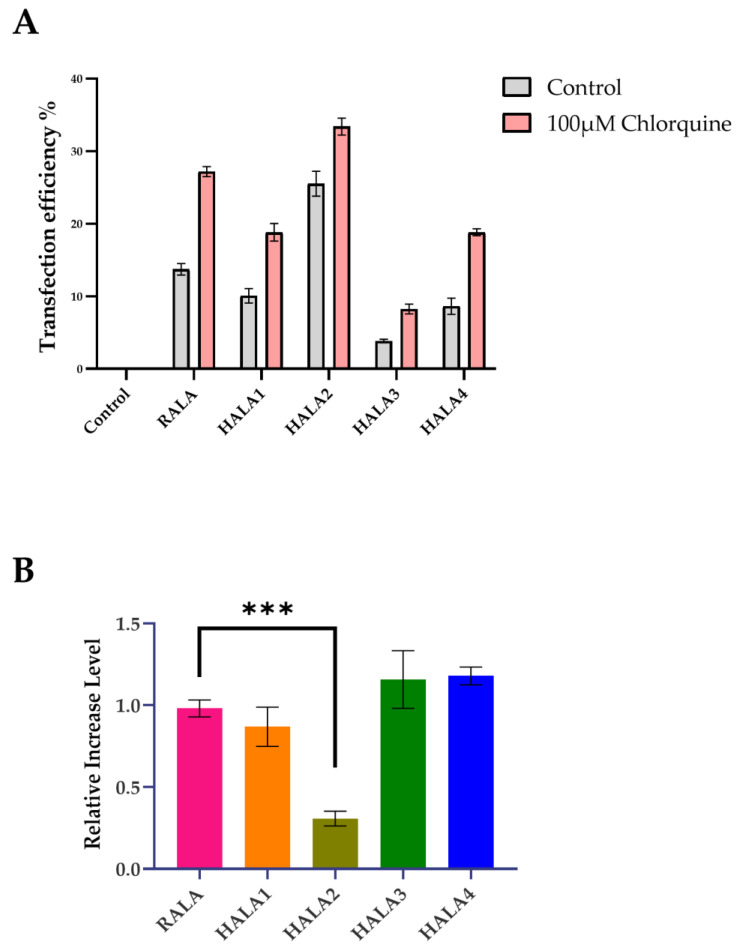
Endosome escaping evaluated by chloroquine. (**A**) The transfection efficacies of the A549 cells transfected with various nanoparticles with (pink) and without (gray) the presence of chloroquine. (**B**) The relative increase levels of transfection efficacies by various nanoparticles. *** Indicates *p* < 0.001.

As shown in Figure 7, HALA2 had a minor increase in transfection efficacy upon the addition with chloroquine, while the rest of the peptides including RALA had a significant increase in transfection efficacy, which was nearly 1-fold (Figure 7A,B). These results reconfirmed that the replacement by histidine in our research increased the endosomal escaping in HALA1 and HALA2. However, HALA3 and HALA4 showed no extra endosomal escaping abilities, presenting a large transfection efficacy increase upon the addition of chloroquine. Furthermore, surprisingly, HALA3 and HALA4 presented similar high values of relative increase in the level of transfection efficacies. A possible explanation for this might be the low transfection efficacies performed originally in the A549 cell line by HALA3 and HALA4.

**Figure 8:**
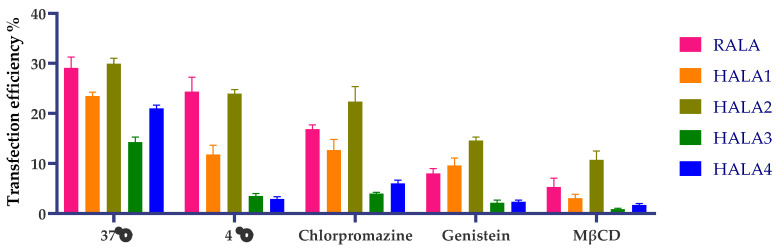
Transfection efficacies incubated under different temperature or with various endocytosis inhibitors.

Next, we intended to discover the mechanism of nanoparticle transfection by RALA and HALA series peptides. The transfection results were also quantified by flow cytometry analysis. We firstly investigated whether this process required energy supplement. A 4 °C incubation environment was employed to suppress the cell metabolism to reduce the energy supply. The results presented that the transfection rates were plunged, indicating that the transfection requires energy supplement (Figure 8).

In the second stage, we intended to use a couple of inhibitors of endocytosis of different pathways to investigate the mechanism of endocytosis of nanocomplexes. As showed in the results, cell transfection efficacies were reduced with all endocytosis inhibitors. Considering this outcome, we assumed that there might be not only one endocytosis pathway involved, although we tend to infer one main pathway for these nanocomplexes. Cells incubated with methyl-β-cyclodextrin (MβCD), a detergent which can remove cholesterol from the plasma membrane and that is capable of inhibiting several pathways including lipid rafts/cholesterol-enriched microdomains/caveolae, unsurprisingly demonstrated the highest inhibition efficacy, which provided strong evidence of our hypothesis that there is more than one pathway functioning in the process of transfection (Figure 8). Subsequently, cells incubated with chlorpromazine, which inhibited the clathrin mediated endocytosis, were also presented results of reduction, yet not as severe as MβCD. Upon the addition of genistein, which is capable of inhibiting caveolae mediated endocytosis, the cells presented a similar lessened trend to MβCD. In the meantime, by placing the previous results of inhibitors according to various peptides, another outcome was shown to us: the HALA2 peptide presented the least influence caused by any inhibitor (Supplementary Figure S2). These results might be due to the efficient endosomal escaping ability of HALA2.

## 3. Discussion

Development of high efficacy and safe CPPs are critical for gene therapy [16,27,28]. Arginine is important for cellular internalization and histidine is helpful for endosomal escaping during gene delivery [29,30]. With this in mind, in this study, we have successfully developed a series of robust CPPs called HALA, by modulation of the histidine and arginine ratio in a model peptide RALA. Among which, HALA1 and HALA2 demonstrated efficient endosomal capacity, and HALA2 further demonstrated superior cell transfection ability compared to the model peptide. These novel peptides possess high potential in gene therapy application and our design strategy opens a new avenue for improving the transfection efficacy of current designed peptides.

Considering the basic features of CPPs, one should be able to firstly condense the nucleic acid cargo and preserve its completeness while forming nanoparticles. However, the histidine replacements of arginine presented on HALA series peptides in this study reduce the number of positive amino acids, and the arginine might lead to the condensed ability reduction due to its electrostatic interaction. Therefore, we employed a gel retardation assay to analyze the basic factor of CPP on RALA and part of the HALA series peptides. According to the result, we found that with two or three arginine replacements, the condensation ability for the gene cargo of HALA peptides showed little change. All the peptides presented with great condensation ability both in pDNA with 5010 bp and siRNA with only 24 bp, indicated that the HALA peptides are able to condense the gene cargo regardless of the length of it, representing that the HALA series peptides have the potential for becoming a gene delivery platform for multiple kinds of genes.

Particle size and charge of nanoparticles were known to be a critical factors for cellular uptake, and efficient cellular uptake would only occur with submicron positively charged particles and suitable size [31]. Here in, we employed Dynamic Light Scattering (DLS) and laser Doppler velocimetry to measure the mean hydrodynamic particle size and zeta potential of the nanoparticles by pDNA complexed with RALA and HALA series peptides. The results indicated an appropriate size and charge of nanoparticles, which were in the suitable range.

An efficient functioning platform for gene delivery requires to be non- or low-cytotoxic. Therefore, we employed the Cell Counting Kit-8 (CCK-8) to evaluate the cell viability after regular transfection process. The results indicated that HALA peptides are cytotoxic free at N:P ratios 1 to 12, indicating basically no impact to host cells. This result is similar to other CPPs, therefore again proving that CPPs possess the potential of clinic application owing to their safety and efficiency.

Next, three cell lines were employed to be the transfection target cells for investigating the transfection ability of various peptides. RALA presented great efficacy as predicted in both HeLa and HEK-293T, although A549 was presented as a lesser one. As for the novel HALA series peptides designed by us, the outcomes were various in different specific peptides. HALA2, which got two arginine replaced by histidine on the C-terminal, presented a particularly great transfected efficacy. On HeLa and HEK-293T cells, transfection efficacies of HALA2 were similar to the RALA peptide. Yet, performing on A549, the percentage of EGFP positive cells in HALA2 group was almost two times compared with RALA. This result indicated to us that HALA2 could be considered as a promising gene vehicle for gene delivery. Yet, we also observed the less transfection results from HALA3 and HALA4 groups, which we believed was owing to the extra histidine replacement in the sequence, leading the condensation ability to be reduced while not presenting on the previous gel retardation assay. More cell lines, including normal and tumoral of various origin, should be tested in further experiments for more accuracy outcomes.

When we collected all the data of thev transfection of pDNA in vitro mentioned above, we found an interesting trend demonstrated in all cell lines. HALA2 always presented a higher transfection efficacy compared with HALA1. Similar results were also detected on the peptides of HALA4 and HALA3. Considering the difference of these various peptides, the results indicated that the position of the replaced histidine on the peptide sequence is also a critical factor for developing a novel cell penetrating peptide, N-terminal replacement compared to C-terminal replacement on the peptide sequence will cause a larger impact on the transfection ability, reducing the efficacy of transfection, comparing to the original peptide. Collectively, we could draw the conclusion that the number of histidine replacements and the positions on the peptide sequence are two main factors contributing to the function of CPPs.

Next, we used siRNA-GAPDH as a siRNA cargo to investigate the gene silencing ability of the HALA series peptides and RALA peptide. From the qRT-PCR results we discover that HALA2 is indeed an efficient gene delivery platform for both pDNA and siRNA, which presented the highest gene silencing rate compared to other peptides. As shown from the Western blotting results, even when comparing two commercially available transfection reagents, Lipofectamine^®^ 2000 and Lipofectamine^®^ 3000, HALA2 presented a similar silencing level to Lipofectamine^®^ 3000, which confirms again that HALA2 could be a safe and efficient vector for further application. Yet, more cell lines should be tested by the siRNA-HALA series peptide complex for more comprehensive data. This job is under consideration in our future work plan.

Considering the function of histidine that we applied in this study on the sequence of CPPs, we assumed that the HALA series peptides are all able to acquire an enhanced endosomal escaping capability. Therefore, we employed a known endosomal disruptor chloroquine, which is capable of damaging the formation process of endosome by inhibiting its acidification. As the results showed, HALA1 and HALA2 presented the less relative increase transfection level compared to RALA, yet the HALA3 and HALA4 interestingly presented an increase. A possible reason of this result could be explained by the poor transfection efficacy performed by HALA3 and HALA4 originally on the A549 cell line. While comparing HALA2 and HALA1, HALA2 presented a lesser increase in the result, which indicated to us again that the replaced histidine on the C-terminal of the RALA sequence is the best match with our assumption. Therefore, we believed that the C-terminal replacement of histidine could maximumly enhance the endosomal escaping ability.

Many works have been done by previous researchers for investigating the mechanism of the nanoparticles, consisted by CPPs and gene cargo, crossing the cell membrane. Most studies believed the process is mostly by endocytosis, which requires energy [32]. Therefore, we created a 4 °C incubational environment for cells which were about to be transfected, trying to inhibit the cell metabolism to cut down the supplement of energy by interfering with the functional temperature. The results unsurprisingly provided the significant evidences to us which indicated that the crossing membrane process indeed requires energy supplement.

Next, we employed several inhibitors related to various endocytosis pathways for a preliminary study of the crossing membrane mechanism of HALA peptides. MβCD was the first inhibitor to be investigated with the transfection process. The results presented that all peptide transection efficacies were decreased. Further experiments incubated with chlorpromazine also presented a result of decrease, but not as much as MβCD. In the group incubated with genistein, the cells presented a similar trend to MβCD. By the results, we can infer that the main endocytosis mechanism of the nanoparticles mentioned in this study is caveolae mediated endocytosis, and it can be inhibited by both MβCD and genistein. Yet there were also other endocytosis pathways that existed, as it was not fully suppressed by these two inhibitors. Further experiments of endocytosis inhibitors on other cell lines are also required for a more precise outcome.

Another interesting outcome drawn by the previous mentioned data is that we realized that the HALA2 peptide presented the least influence caused by any of the inhibitors. A possible explanation for this outcome might be due to the efficient endosome escaping ability of HALA2. With the help of histidine, nanoparticles contain certain numbers of pDNA which reach a certain threshold and are capable of escaping from the endosome efficiently and release pDNA for the upcoming EGFP expression. However, the HALA3 and HALA4, which had three arginine replaced by histidine, presented significant influence by all the inhibitors. These outcomes could be explained by the low transfected rate in the A549 cell line. We would like to put this part into our further research plan.

Other effective CPPs would be employed to study further, for providing evidence that histidine replacements on CPPs is a universal method for enhancing the endosome escaping ability on existing peptides.

Our data demonstrates the impact of histidine replacements on CPPs, pointed out two main factors: that the number of histidine replacements and positions on the peptide sequence (N-terminal or C-terminal) contribute to the final outcomes. The results indicated the merits of HALA series peptides in comparison with the original peptide RALA, through enhancement of CPPs endosome escaping ability. Furthermore, we discovered a novel CPP, HALA2, as indeed a highly effective gene delivery platform for both DNA and RNA in vitro, demonstrating a better efficacy on the HEK-293T and A549 cell lines compared with RALA and Lipofectamine^®^ 3000, indicating a potential translation to clinical application.

## 4. Conclusions

Considerable data indicate that replacement of arginine on the RALA peptide sequence by histidine with the suitable number and position of the C-terminal is an effective method to develop novel CPPs by enhancing the endosome escaping ability. We also evaluated a novel CPP, HALA2, which presented a great nucleic acid cargo condensation ability and was cytotoxic free, as indeed a highly effective gene delivery platform for both DNA and RNA in vitro. HALA2 demonstrated a better pDNA and siRNA transfection efficacy compared with other peptides, indicated a promising gene delivery platform for clinical application.

## 5. Materials and Methods

### 5.1. Preparation of Peptides-Nucleic Acid Nanocomplexes

The RALA peptide and HALA series peptides were produced by Jiangsu Ji Tai peptide Industry Science and Technology Co, Ltd. (Yancheng, China), supplied as a desalted lyophilized powder. Then sterilized ddH_2_O (Milli-Q, Merck KGaA, Darmstadt, Germany) were used to dissolve the powder and stored in small aliquots at −20 °C. The pCMV-EGFP plasmid contained the Enhanced Green Fluorescent Protein (EGFP) gene under the control of the cytomegalovirus promoter, was purchased from Beyotime (Shanghai, China #D2626). The Human siRNA-GAPDH was purchased from GenePharma (Suzhou, China #A08008), 5′Cy3 modified non-target siRNA and regular non-target siRNA was purchased from Ribobio (Guangzhou, China). The sequence of siRNA-GAPDH is 5′-UGACCUCAACUACAUGGUUTT-3′.

The pCMV-EGFP plasmid was firstly transferred into *Trans*1-T1 Phage Resistant Chemically Competent Cell from Transgen (Beijing, China #CD501-02), then amplified from overnight bacterial cultures in Lysogeny Broth and obtained by alkaline lysis and purified using a plasmid Maga kit under endotoxin-free conditions (Magen, Guangzhou, China, #P1114-2). The purified plasmid was diluted in TE buffer pH 8.0 to 1mg/mL and quantified by ultraviolet (UV) ray absorption at 260 nm by using NanoDrop (Thermo Fisher Scientific, Waltham, MA, USA) to 1mg/mL and stored in small aliquots at−20 °C.

Peptides-nucleic acid nanoparticles were prepared at various N:P ratios, the molar ratio of positively charged nitrogen atoms in the peptide to negatively charged phosphate in the nucleic acid backbone, in the range of 1 to 12. All aminos with positive charge from various peptides were taken into account for the N:P ratio. The required amount of nucleic acid-peptide complexes was prepared in ddH_2_O (Milli-Q, Merck KGaA, Darmstadt, Germany) with the order of plasmid or siRNA solution firstly added in ddH_2_O, then transported with the right amount of peptide solution in it. The final volume was adjusted to 50 μL. The complexes were mixed immediately by using a pipet, and were allowed to stay at room temperature for 30 min for incubation. All nanocomplexes were used immediately after their preparation.

### 5.2. Cell Culture

The HeLa cell line, A549 cell line, and HEK-293T cell line were kindly provided by Stem Cell Bank, Chinese Academy of Sciences (Shanghai, China). Routine tests confirmed these cells were *Mycoplasma*-free. They were used as model cells for siRNA and pDNA transfection in this study. All cell lines were maintained as monolayers in high glucose Dulbecco’s Modified Eagle’s Media (DMEM) (Gibco, Waltham, MA, USA) supplemented with 1% fetal bovine serum (FBS) (Gibco, Waltham, MA, USA) and 1% penicillin–streptomycin solution (Invitrogen, Waltham, MA, USA). When the cells reached 80% confluency approximately, they were passaged. All cell lines were cultured under the standard cell culture conditions with the atmosphere of 5% CO_2_ and 90% humidity at 37 °C.

### 5.3. Cell Transfection

For pDNA transfection, cells were seeded in 24-well plates at a density of 5 × 10^4^ cells per well and waited for adhesive overnight at 37 °C with 5% CO_2_. Before the transfection, the original culture media were replaced by 300 μL Opti-MEM^TM^ Reduced Serum Medium (Gibco, Waltham, MA, USA) for 1 h. For pDNA transfection, 50 μL of nanoparticles solution containing 500 ng pDNA was added to the wells and gently mixed with the Opti-MEM. As for siRNA-GAPDH transfection, 50 μL of nanocomplex solution was prepared by calculation of the concentration of siRNA in final culture media to be 40 nM. Cells were then incubated with the nanocomplex for 6h under the condition of 37 °C with 5% CO_2_, before being replaced with the fresh original complete media.

Cell transfection was repeated with changing the culture environment in 4 °C, or with the addition of either 100 μg/mL chloroquine (Aladdin, Shanghai, China), 5 mg/mL Methyl-β-cyclodextrin (MβCD) (Aladdin, Shanghai, China), 10 mg/mL Chlorpromazine (Aladdin, Shanghai, China) or 10 mg/mL Genistein (Aladdin, Shanghai, China) to investigate the mechanism of endocytosis and endosomal escape of the nanoparticles. Cells transfected with the addiction of various inhibitors or incubated under 4 °C were analyzed by flow cytometer.

### 5.4. Size and Zeta Potential Analysis of Nanocomplexes

Nanocomplexes were prepared as previous described in various N:P ratio at a range of 1 to 12. The nanoparticles solution was diluted 20 times to 1 mL by ddH_2_O for further measurement. A Malvern Zetasizer ZS 3000 (Malvern Instruments, Worcestershire, UK) were used to measure the mean hydrodynamic particle size of nanoparticles dispersed in ddH_2_O by Dynamic Light Scattering (DLS) at 25 °C and zeta potential were using Laser Doppler Velocimetry.

### 5.5. Gel Electrophoresis of Peptides-Nucleic Acid Nanocomplexes

Nanocomplexes were prepared as previous described at a range of N:P ratios, the molar ratio of positively charged nitrogen atoms in the peptide to negatively charged phosphate in the nucleic acid backbone, from 0 to 12. After mixed with 2 μL loading dye (Thermo Fisher Scientific, Waltham, MA, USA), samples of all groups were electrophoresed through a 1% agarose gel containing GelRed Nucleic acid gel stain (Accurate Biology, Changsha, China #11918) within Tris-acetate (TAE) running buffer at 100 V for 60 min. The siRNA samples were mixed with 2 μL loading dye (Thermo Fisher Scientific, Waltham, MA, USA) before electrophoresed through a 20% polyacrylamide Gel (Thermo Fisher Scientific, Waltham, MA, USA) within Tris-borate-EDTA (TBE) buffer at 120 V for 30 min. Mobility of nucleic acid was visualized using a ChemiDoc Touch (Bio-Rad, Hercules, CA, USA).

### 5.6. Fluorescence Microscopy

In order to facilitate qualitative analysis of pCMV-EGFP expression which correlated to transfection efficiency, cells after transfection were imaged 24 h following transfection under fluorescein light, using a Leica DMI8 (Leica, Wetzlar, Germany) to visualize.

### 5.7. Quantification of Peptide Uptake Efficiency by Flow Cytometric Analysis

The amount of uptake efficiency of peptide was quantified by a flow cytometric analysis performed by CytoFLEX (Beckman Coulter, Brea, CA, USA). After transfection processes as described previously, cells were allowed to culture for another 2 days. PBS was used to washed gently twice and the cells were digested with Trypsin (Gibco) for 3 min before collected in 1.5 mL microcentrifuge tubes. Cells were collected by centrifuging under the condition of 3500 rpm/min, and washed with PBS one time before resuspended with FACS buffer. Data were analyzed by using the FlowJo V10 software (BD Life Sciences–FlowJo, Ashland, OR, USA).

### 5.8. CCK-8 Assays

Cytotoxicity was estimated using a Cell Counting Kit-8 (CCK-8) (Beyotime, #C0037). Cells were seeded in 96-well plates with a density of 5 × 10^3^ cells per well and kept in an incubator overnight for adhesive. A total of 60 μL Opti-MEM^TM^ with nanoparticles, prepared as previous described in various N:P ratios, was added in for 6 h transfection, before replacing the media with fresh one. Cells were allowed to culture for 24 or 48 h in the incubator. Then, PBS was used to wash the well twice before adding the CCK-8 working solution with 90% fresh media and 10% CCK-8 stock solution. Plates were placed in the incubator for 2 h before measuring the absorbance of each well at 450 nm by using a Synergy H1 Hybird Reader (BioTek Instruments, Winooski, VT, USA).

### 5.9. qRT-PCR

A549 cells were seeded in a 6-well plate with a density of 2 × 10^5^ cells per well, and allowed for culturing overnight for adhesion before transfection. The transfection processes was as previous described. GAPDH gene expression in A549 cell line was evaluated 72 h after transfection by firstly extracted the total RNA by using TRIzol reagents (Invitrogen, USA), then the reverse transcription of first-stand cDNA was using a PrimeScript RT reagent kit (Takara, Kusatsu, Shiga, Japan) following protocols recommended by the manufacturer. Real-time quantitative polymerase chain reaction analyses for mRNA of GAPDH and actin were performed by using Hieff UNICON Universal Blue qPCR SYBR Green Master Mix (YEASEN, Shanghai, China). The sequences of oligonucleotides used are as follows:

- Human GAPDH-F: 5′-aggtcggagtcaacggatttg-3′
- Human GAPDH-R: 5′-gtgatggcatggactgtggt-3′
- Human Actin-F: 5′-ctccatcctggcctcgctgt-3′
- Human Actin-R: 5′-gctgtcaccttcaccgttcc-3′

### 5.10. Western Blot

A549 cells were firstly seeded in a 6-well plate with a density of 2 × 10^5^ cells per well, and allowed for culturing overnight for adhesion before transfection. Total protein in A549 cell line was evaluated 96 h post-transfection as previous described by using lysis buffer. Cell lysis solution were centrifuged at 12,000× *g* at 4 °C for 15 min. A bicinchoninic acid protein assay kit (Thermo Fisher Scientific) was used to analyze the protein concentrations. The equal amounts of 20 μg of the protein were separated by 10% sodium dodecyl sulfate polyacrylamide gel electrophoresis gel and transferred to a nitrocellulose membrane. The membranes were then blocked with 5% nonfat dry milk in Tris-buffered saline and incubated with the following primary antibodies overnight at 4 °C, Anti-β-Actin Mouse Mab (1C7) (Abcam, Waltham, MA, USA) and Anti-GAPDH Mouse Mab (2B5) (Abcam, Waltham, MA, USA) with the concentrations recommended by the manufacturer. After being washed with Tris-buffered saline with 0.1% Tween-20 detergent (TBST) buffer, the membranes were incubated with Rabbit anti-Mouse IgG (H + L) Cross-Adsorbed secondary antibody, the Alexa Fluor 488 (Invitrogen, Waltham, MA, USA), with the concentrations recommended by the manufacturer for 2 h at room temperature. Images of the membranes were visualized and captured using a ChemiDoc Touch (Bio-Rad, Hercules, CA, USA).

## 6. Statistical Analysis

Statistical analysis was performed with the use of Prism 5.0 (GraphPad Software, Inc., La Jolla, CA, USA). Statistically significant data differences were calculated using the one-tailed unpaired *t*-test or one way analysis of variance followed by Dunnett’s post hoc test with a *p*-value of ≤0.05 considered as significant. Three independent experiments were performed for each analysis and all data results are presented as mean ± SD (n = 3), unless otherwise specified.

## Figures and Tables

**Table 1:** The sequence of RALA and HALA peptides. The sequence of the HALA series peptides designed by replacing the arginine by histidine on RALA peptides. Various HALA peptides were named by the variety of the number of histidine replacements and their positions in the sequence. Positive charged amino acid numbers were also presented.

Peptide	Sequence	Positive Charged Amino Acid Number
RALA	WEARLARALARALARHLARALARALRACEA	7
HALA1	WEAHLAHALARALARHLARALARALRACEA	5
HALA2	WEARLARALARALARHLARALAHALHACEA	5
HALA3	WEAHLAHALAHALARHLARALARALRACEA	4
HALA4	WEARLARALARALARHLAHALAHALHACEA	4

## Data Availability

The data of this study are available from the corresponding author on reasonable request.

## Supplementary Materials

The following are available online at https://www.mdpi.com/article/10.3390/ma14164674/s1, Figure S1: Cellular uptake and transfection efficacy of the nanoparticles. (A) Flow cytometry was employed for investigating the uptake efficiency of nanoparticles complexed by RALA and HALA series peptide with 5′Cy3 modified non-target siRNA. A group of non-treated cells were used as control. (B,C,D) flow cytometry was employed for evaluating the transfection efficiency of nanoparticles complexed by RALA and HALA series peptide with pCMV-EGFP plasmid DNA in HeLa cells (B), HEK-293T cells (C) and A549 cells (D). A group of cells treated with bare pDNA solution containing 1 μg pDNA per well of 24-well plate were used as control, Figure S2: Transfection efficacies of nanocomplexes generated by pDNA and different peptides incubated under 4 °C or with various endocytosis inhibitors under 37 °C, grouped by different peptides.
