# Supplementary material for: Development and Characterization of High Efficacy Cell-Penetrating Peptide via Modulation of the Histidine and Arginine Ratio for Gene Therapy
Source: Materials (Basel). 2021 Aug 19;14(16):4674. doi: 10.3390/ma14164674 (PMC8399742; doi:10.3390/ma14164674)
Supplement: Supplementary file 1 [file materials-14-04674-s001.zip › materials-1290062-supplementary.pdf]

materials

MDPI

Supplementary

Development and Characterization of High Efficacy Cell-Penetrating Peptide via Modulation of the Histidine and Arginine Ratio for Gene Therapy

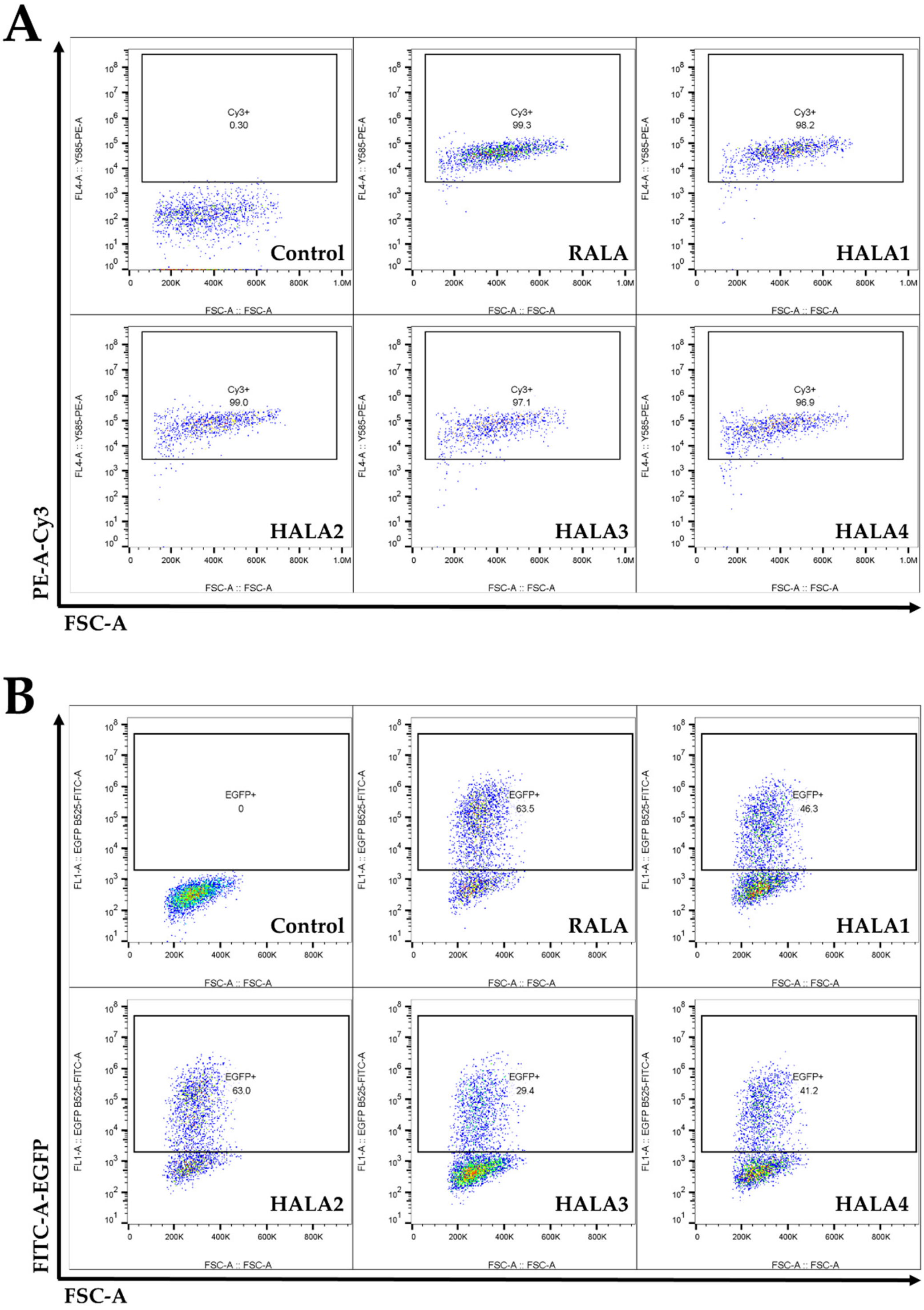

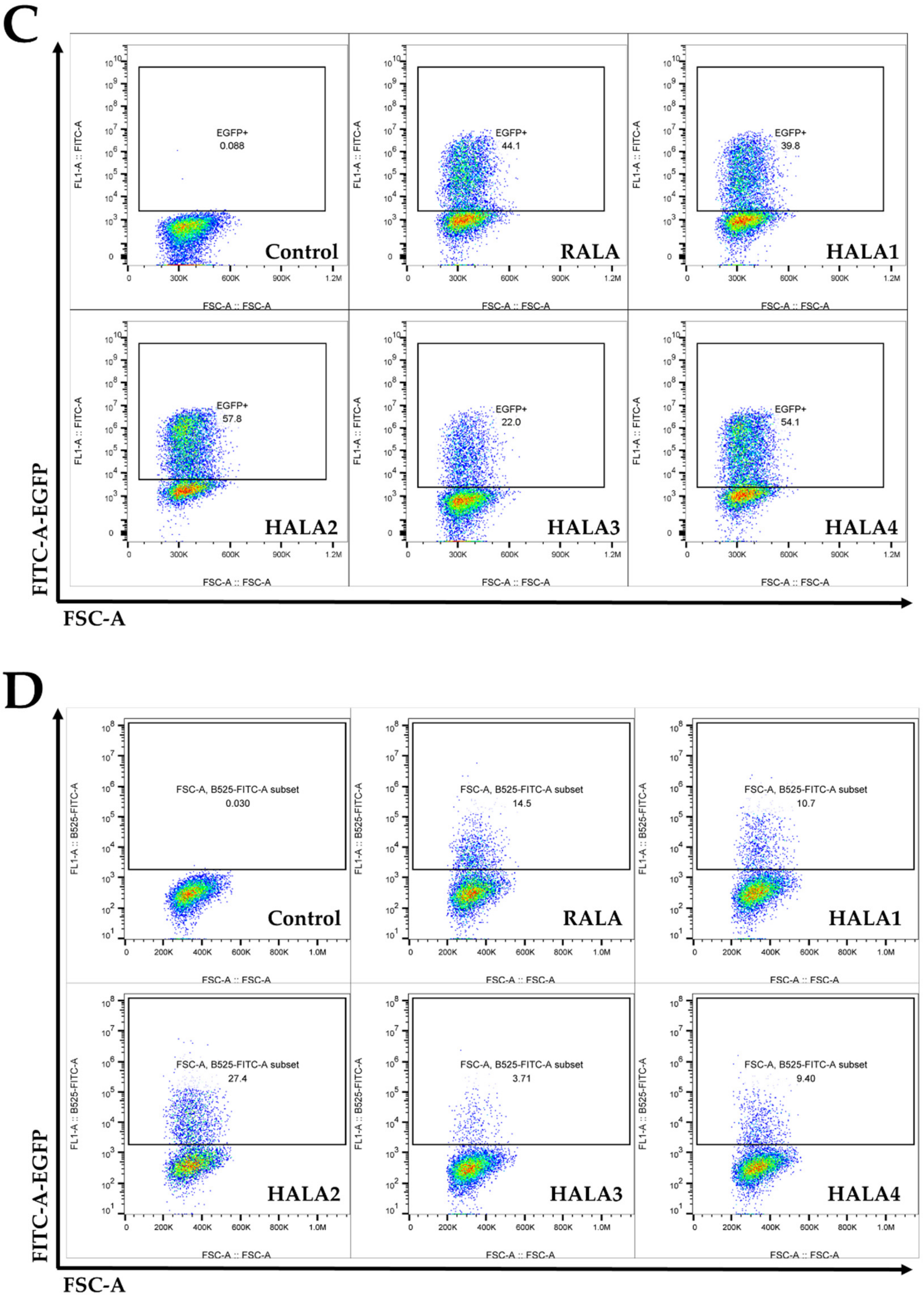

Figure S1. Cellular uptake and transfection efficacy of the nanoparticles. **(A)** Flow cytometry was employed for investigating the uptake efficiency of nanoparticles complexed by RALA and HALA series peptide with 5′Cy3 modified non-target siRNA. A group of non-treated cells were used as control. **(B, C, D)** flow cytometry was employed for evaluating the transfection efficiency of nanoparticles complexed by RALA and HALA series peptide with pCMV-EGFP plasmid DNA in HeLa cells **(B)**, HEK-293T cells **(C)** and A549 cells **(D)**. A group of cells treated with bare pDNA solution containing 1 μg pDNA per well of 24-well plate were used as control.

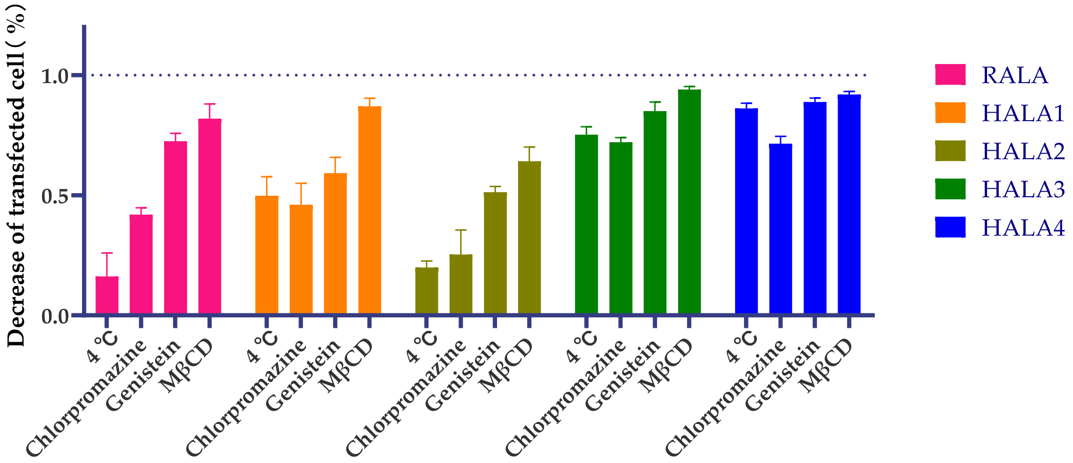

Figure S2. Transfection efficacies of nanocomplexes generated by pDNA and different peptides incubated under 4 °C or with various endocytosis inhibitors under 37 °C, grouped by different peptides.
